# Surgical Treatment for Chronic Pancreatitis With a Normal-Sized Pancreatic Head and a Dilated Duct: Frey or Extended Partington Procedure?

**DOI:** 10.7759/cureus.61881

**Published:** 2024-06-07

**Authors:** Dai Nga Mai, Quoc Vinh Nguyen, Minh Tri Phan, Tien My Doan

**Affiliations:** 1 Hepatobiliary and Pancreatic Surgery, Cho Ray Hospital, Ho Chi Minh, VNM; 2 Surgery, University of Medicine and Pharmacy at Ho Chi Minh City, Ho Chi Minh, VNM

**Keywords:** normal-sized head of the pancreas, inflammatory mass in the head of the pancreas, extended partington procedure, frey procedure, chronic pancreatitis

## Abstract

Background: Surgical drainage for chronic pancreatitis patients with a normal-sized pancreatic head remains controversial. Both Frey and extended Partington procedures could be used, but the level of evidence is weak.

Method: The object of this prospective cohort study was to assess the mid-term results concerning pain, quality of life, and pancreatic function of surgical drainage (Frey or extended Partington procedure) in patients with painful chronic pancreatitis and a normal-sized pancreatic head.

Results: Fifty-nine patients (Frey procedure: 14 cases; extended Partington procedure: 45 cases) were enrolled in the study with a median length of follow-up of 16 months. The effective and complete pain relief rate was 85% and 58%, respectively. The Izbicki score decreased from 53.4 preoperatively to 8.8 postoperatively. The general 12-Item Short Form Health Survey (SF-12) score increased from 45.2 to 75.4. The pancreatic insufficiency did not change significantly postoperatively. At three months after surgery, the complete pain relief and Izbicki score were more favorable in the Frey group than in the extended Partington group.

Conclusion: Both Frey and extended Partington procedures resulted in excellent pain relief and quality of life improvement and did not worsen pancreatic function. The Frey procedure could yield a more favorable result in the early postoperative period.

## Introduction

Chronic pancreatitis is an inflammatory disease of the pancreas in which the normal parenchyma is gradually replaced with fibrotic and calcified tissue [1]. Pain is the most suffered symptom of this disease. The pain mechanism is thought to be perineural inflammation due to an enlarged pancreatic head or intraductal hyper-pressure due to a dilated main pancreatic duct [2]. The pacemaker of chronic pancreatitis is presumably at the head of the pancreas, which comprises the majority of pancreatic parenchyma [3]. Surgery aiming to exclude the enlarged pancreatic head or relieve the ductal pressure is the choice of treatment [4]. In most Asian countries, the enlarged pancreatic head is occasionally seen; surgical drainage is the most used treatment for chronic pancreatitis [5].

Partington procedure has been used for a long time for patients with a dilated duct. However, over half of the patients who underwent this procedure acquire recurrent pain attacks; some even require reoperation to redrain the duct [6]. The original Partington procedure leaves the head of the pancreas intact; therefore, some stricture ducts or stones in this area may cause a problem later [7]. To overcome this pitfall, extended pancreatic duct drainage such as Frey or extended Partington procedure are recommended [6,8].

Frey procedure combines limited resection of the pancreatic head with drainage of the main pancreatic duct [9]. This procedure had been used only for patients with an enlarged pancreatic head. However, Ho and Frey [10] suggested using it even for patients without an inflammatory mass in the head, with the concept that the stricture ducts, which often contain stones, may lie deep into the parenchyma of the pancreatic head and can be excluded only by coring out of the head.

O’Neil and Aranha [11] modified the original Partington procedure, in which an additional duct incision into the head of the pancreas ensures complete duct drainage and removal of stones. This modification is called the extended Partington procedure. Given the disadvantages of the original Partington procedure in pain relief, a consensus was made to encourage the use of both Frey or extended Partington procedure for chronic pancreatitis with a normal-sized pancreatic head and a dilated duct [8]. However, the level of evidence is still weak. Furthermore, studies that directly compare these two surgical techniques are still lacking. We conducted this study to assess the mid-term results of both Frey and extended Partington procedures concerning pain relief, quality of life, and pancreatic function and partly make some comparisons of these two procedures if possible.

## Materials and methods

Study design and population

After approval of the study by the medical ethics committee of the University of Medicine and Pharmacy at Ho Chi Minh City, coded 2186/ĐHYD, on February 3, 2021, we conducted this study in the Department of Hepatobiliary and Pancreatic Surgery, Cho Ray Hospital, Ho Chi Minh City, Viet Nam. This is a prospective cohort study that comprises painful chronic pancreatitis patients with a normal-sized pancreatic head and a dilated duct. We used both Frey and extended Partington procedures for this population in a non-randomized fashion. Written informed consent was obtained from all patients before enrollment.

Inclusion and exclusion criteria

Patients were diagnosed with chronic pancreatitis according to the M-ANNHEIM definite criteria [12]. The pain lasted for at least three months, requiring a minimum of two weeks of weak opioids (tramadol or codeine) or constant for a month, unrelievable with weak opioids. A dilated pancreatic duct must exist on imaging (computed tomography or magnetic resonance imaging). Patients who satisfied the above criteria were eligible for the study. Patients with an enlarged pancreatic head, patients who met surgical contraindications such as American Society of Anesthesiologists (ASA) class IV, severe portal hypertension, patients with pancreatic cancer, and patients with a length of follow-up inferior to three months were excluded from the study. A dilated duct is defined as the largest diameter ≥ 5mm [13]. An enlarged pancreatic head is defined as the largest diameter ≥ 40mm [13].

Surgical techniques

Extended Partington Procedure

After the abdomen is entered, an extensive Kocher maneuver and an incision of the gastrocolic ligament are performed to expose all parts of the pancreas. The mesentery of the transverse colon must be taken down, and the superior mesenteric vein at the inferior border of the pancreas must be seen to expose entirely the uncinate process. The pancreatic duct is incised from 1-2cm from the tip of the tail to the neck of the pancreas. Before extending the duct incision into the head, parenchymal sutures at the inner border of the duodenum and the right border of the superior mesenteric vein are performed. The anterior pancreatoduodenal arcade must be ligated on both sides of the duct. The duct is then incised into the head, and the uncinate process of the pancreas up to 1cm from the duodenum. All stones are removed as much as possible. A limited wedge resection along the duct incision can be carried out if the difficulty of stone removal is met. If this procedure cannot entirely retrieve stones, the Frey procedure should be performed.

Frey Procedure

**Figure 1:**
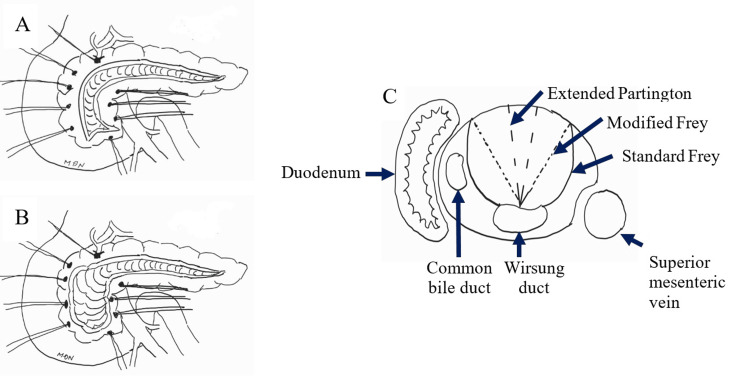
Illustration of extended pancreatic duct drainage procedures (A) Frontal view of extended Partington procedure: the duct incision is carried out into the Wirsung and the uncinate process duct to a point proximal to the duodenum. (B) Frontal view of Frey procedure: the head and the uncinate process of the pancreas are cored out, leaving a pancreatic rim along the duodenum and the superior mesenteric vein. (C) A cross-section of the pancreatic head shows the difference between the three procedures: a straight line coming from the pancreatic anterior surface into the lumen of the duct in the extended Partington procedure, an oblique line coming from the pancreatic anterior surface at the border of duodenum and superior mesenteric vein into the lumen of the duct in the modified Frey procedure, and a convex curve coming from the pancreatic anterior surface at the border of duodenum and superior mesenteric vein into the lumen of the duct in the standard Frey procedure. Image Credits: Dai Nga Mai.

The Frey procedure is performed in cases of densely fibrotic parenchyma in the pancreatic head or the inability to complete stone removal by pure duct incision. All the preliminary steps are the same as the extended Partington procedure. The parenchymal excision begins from the anterior surface along the inner border of the duodenum and the right side of the superior mesenteric vein. The excision comes posteriorly in a convex curve into the lumen of the main pancreatic duct. A thin layer of pancreatic parenchyma posterior to the duct is left intact. The remnant parenchymal rim along the duodenum and the superior mesenteric vein is about 1cm thick. A modified Frey procedure can be performed, given the fear of perforation into the posterior surface of the pancreas. The anterior surface excision comes posteriorly in an oblique line into the lumen of the duct instead of a convex curve as in the standard Frey. This modified procedure was used limited in our early cases. The standard Frey, modified Frey, and extended Partington procedures are illustrated in Figure 1. After extended pancreatic duct drainage is accomplished, a longitudinal pancreaticojejunostomy in a Roux en Y fashion is performed.

Definition of outcomes

The pain was assessed using the Izbicki score, a tool comprising four aspects: frequency, the requirement for analgesic use, intensity using the Visual Analog Scale (VAS), and inability to employ [14]. Effective pain relief was defined as a reduction of ≥ 50% of the baseline Izbicki score [15]. Complete pain relief was defined as an absolute Izbicki score ≤ 10 [15]. The 12-Item Short Form Health Survey (SF-12) assessed the quality of life. This tool is reliable, concise, brief, and easy to use. The SF-12 comprises 12 questions regarding two health indices: the physical component summary (PCS) and the mental component summary (MCS) [16,17].

Pancreatic function was assessed by exocrine and endocrine sufficiency. Endocrine insufficiency was confirmed in the presence of any criteria: fasting serum glucose ≥ 128 mg/dl, any serum glucose ≥ 200 mg/dl, or the requirement of using glycemic control agents (oral drugs or insulin). Given the lack of fecal tests for fat or pancreatic enzymes, we defined exocrine insufficiency as steatorrhea. Other manifestations such as weight loss, nausea, anorexia, diarrhea, weight, and body mass index (BMI) were also included.

Statistical analysis

The quantitative variables were demonstrated as means with standard deviation (SD) if they were normally distributed or medians with range if they were nonparametric. The categorical variables were demonstrated as number of patients with percentage. The Shapiro-Wilk test checked the normal distribution of the variables. The Student's t-test checked the difference between two normally distributional variables. The Mann-Whitney u-test checked the difference between two non-parametric variables. When appropriate, the chi-square and Fisher exact test checked the difference between the two frequencies.

## Results

Baseline characteristics

From November 2021 to November 2023, 59 patients who met the inclusion criteria and did not meet the exclusion criteria were enrolled in the study. The Frey procedure was performed in 14 cases, whereas the extended Partington procedure was used in 45 cases. Table 1 demonstrates the characteristics of preoperative clinical presentations, pain, quality of life, and morphology. The etiology was considered as alcohol if the patient drank significantly, as cigarette if the patient smoked significantly, as hereditary if there were any of three-generation related family members of the patient diagnosed with chronic pancreatitis, and as idiopathy if no above etiology was found. We did not find any case of hereditary causes among patients. The preoperative pain score was high, and the quality of life was low. The rates of exocrine and endocrine insufficiencies were considerable. All patients presented with stones at the head of the pancreas. Morphological complications such as biliary obstruction and pseudocyst were met considerably. Pseudocyst was met more in the Frey group than the extended Partington group. The pancreatic head was larger in the Frey group than in the extended Partington group. No other significant difference was seen.

**Table 1:** Baseline characteristics ^a^ Statistically significant difference between the Frey and the extended Partington groups, Fisher exact test. ^b^ Statistically significant difference between Frey and extended Partington groups, Student’s t-test. ASA: American Society of Anesthesiologists, VAS: Visual Analog Scale, SF-12: 12-Item Short Form Health Survey, PCS: physical component summary, MCS: mental component summary

Variables	Combined	Extended Partington	Frey
Age	42 (SD: 12)	43 (SD: 13)	40 (SD: 11)
Sex and gender			
Male	47 (80%)	35 (78%)	12 (86%)
Female	12 (20%)	10 (22%)	2 (14%)
Time from onset (months)	48 (range: 1-192)	48 (range: 1-192)	48 (range:1-132)
Etiology			
Alcohol	37 (63%)	27 (60%)	10 (71%)
Cigarette	5 (8%)	5 (11%)	0 (0%)
Idiopathic	17 (29%)	13 (29%)	4 (29%)
ASA Class			
Class I	23 (39%)	20 (44%)	3 (21%)
Class II	34 (58%)	23 (51%)	11 (79%)
Class III	2 (3%)	2 (5%)	0 (0%)
Izbicki score	53.4 (SD: 13.8)	51.9 (SD: 13.4)	58.4 (SD: 9.3)
Frequency	61.7 (SD: 21.3)	59.4 (SD: 29.8)	68.9 (SD: 29.4)
Analgesics use	15 (range: 3-75)	15 (range: 3-15)	15 (range: 15-75)
VAS	80.8 (SD: 14.6)	80 (SD: 14.5)	83.2 (SD: 15.1)
Inability to work	55.5 (SD: 26.7)	53.3 (SD: 27.5)	62.5 (SD: 23.5)
General SF-12 score	45.2 (SD: 21)	45.6 (SD: 20)	41.1 (SD: 24.8)
PCS	41 (SD: 24.3)	41.6 (SD: 23.2)	39.2 (SD: 28.5)
MCS	49.4 (SD: 24.7)	49.5 (SD: 24)	49 (SD: 27.8)
Exocrine insufficiency	18 (31%)	15 (33%)	3 (21%)
Diabetes mellitus	19 (31%)	17 (38%)	2 (14%)
Insulin dependant	10 (17%)	9 (20%)	1 (7%)
Dose of insulin (IU)	22 (SD: 9)	21 (SD: 9)	25
Weight (kg)	52.1 (SD: 8.4)	52.2 (SD: 8)	51.8 (SD: 9.7)
BMI (kg/m^2^)	19.5 (SD: 2.5)	19.9 (SD: 2.3)	18.6 (SD: 3)
Weight loss	44 (75%)	33 (73%)	11 (79%)
Nausea	24 (41%)	17 (38%)	7 (50%)
Anorexia	25 (42%)	20 (44%)	5 (36%)
Diarrhea	13 (22%)	10 (22%)	3 (21%)
Biliary obstruction	8 (14%)	3 (7%)	5 (36%)
Pseudocyst ^a^	11 (19%)	5 (11%)	6 (43%)
Pancreatic concretions			
Head	50 (100%)	45 (100%)	14 (100%)
Parenchyma	33 (56%)	26 (58%)	7 (50%)
Duct	40 (68%)	31 (69%)	9 (64%)
Diameter of pancreatic duct (mm)	10 (range: 5-20)	10 (range: 5-20)	10 (range: 5-15)
Diameter of pancreatic head (mm) ^b^	29.4 (SD: 4.5)	28.7 (SD: 4.5)	31.8 (SD: 3.7)

Operative characteristics and early outcomes

We managed biliary obstruction by choledochojejunostomy in three patients undergoing the extended Partington procedure and by reinsertion of the bile duct into the resection cavity in five patients undergoing the Frey procedure. One case among patients with biliary obstruction had gastrojejunostomy due to gastric outlet obstruction. All pseudocysts were incorporated into the duct incision except one case, which was resected along with a pancreatic tail due to its massive size. The median blood loss was 100 ml (50-100), with no difference between Frey and the extended Partington procedure. One case in the Frey group required blood transfusion intraoperatively. The median operative time was 210 minutes (140-370), with no difference between the two groups.

Five patients developed postoperative complications, which made it a total of 9%, with 4% in the extended Partington group and 21% in the Frey group (p=0.08, Fisher exact test) (Table 2). One patient in the extended Partington group had postoperative adhesive bowel semi-obstruction, which was medically managed successfully. One case in the Frey group had gastrointestinal bleeding presenting by melena, which required rehospitalization and blood transfusion; this patient stopped bleeding and had no further event. One patient in the Frey group had a pancreatic fistula grade B, according to the International Study Group on Pancreatic Surgery, which was managed nonoperatively successfully. Another case in the Frey group developed a chyle leak after four weeks of operation, which was managed successfully by paracentesis and medicine. Only one patient in the extended Partington group was reoperated in the early postoperative course because of postoperative gastrointestinal bleeding unmanageable by conservative treatment; the intraoperative finding was bleeding at the site of the pancreatic head incision; this patient was stable after that and had no further event. No early postoperative deaths occurred.

**Table 2:** Early and mid-term outcomes * There are statistically significant differences between the time of follow-up and the baseline, using the Mann-Whitney u-test. ^a^ There is a statistically significant difference between the Frey group and the extended Partington, using the Mann-Whitney u-test. VAS: Visual Analog Scale, SF-12: 12-Item Short Form Health Survey, PCS: physical component summary, MCS: mental component summary

Variables	Combined	Extended Partington	Frey
Early morbidity	5 (9%)	2 (4%)	3 (21%)
Clavien grading			
Grade I	1 (2%)	1 (2%)	0 (0%)
Grade II	2 (4%)	0 (0%)	2 (14%)
Grade III	2 (4%)	1 (2%)	1 (7%)
Grade IV	0 (0%)	0 (0%)	0 (0%)
Grade V (mortality)	0 (0%)	0 (0%)	0 (0%)
Length of postoperative stay (days)	6 (range: 3-17)	6 (range: 4-8)	7 (range: 3-17)
Follow-up time (months)^a^	16 (range: 3-24)	18 (range: 3-24)	6 (range: 3-21)
Effective pain relief	50 (85%)	37 (82%)	13 (93%)
Complete pain relief	34 (58%)	24 (53%)	10 (71%)
Izbicki score*	8.8 (range: 0-78.8)	8.8 (range: 0-78.8)	8.8 (range: 0-28.3)
Frequency*	25 (range: 0-100)	25 (range: 0-100)	25 (range: 0-25)
Analgesics use*	0 (range: 0-15)	0 (range: 0-15)	0 (range: 0-3)
VAS*	10 (range: 0-100)	10 (range: 0-100)	10 (range: 0-35)
Inability to work*	0 (range: 0-100)	0 (range: 0-100)	0 (range: 0-50)
General SF-12 score*	75.4 (range: 15.8-100)	76.7 (range: 15.8-100)	73.1 (range: 39.6-100)
PCS*	79.2 (range: 8.3-100)	75 (range: 8.3-100)	79.2 (range: 41.7-100)
MCS*	76.7 (range: 16.7-100)	76.7 (range: 16.7-100)	75 (range: 37.5-100)

Mid-term follow-up

At the end of the study, patients were followed for a median of 16 months. The follow-up time in the Frey group was six months, much shorter than the extended Partington group with 18 months (p<0.001, Mann-Whitney u-test). There were three cases of mid-term mortality in which causes were not in direct relation to the operation, including a cerebrovascular accident at five months, a visceral perforation at 11 months, and a flared tuberculous pneumonia at 19 months. One patient continuously suffered pain, which was unrelievable with even strong opioids, and ultimately underwent thoracoscopic splanchnicectomy to control the pain at four months. Two patients were lost to follow-up appointments at six months. Cases of mortality, reoperation due to resistant pain, and loss in follow-up were considered the failure of treatment, which made it 10%. Two patients developed late complications, including an incisional hernia at four months, which required surgical repair, and a recurrent biliary obstruction at 16 months, which required a new biliary derivation of choledochojejunostomy.

Pain and quality of life outcomes

Table 2 demonstrates the pain relief rate, Izbicki score, and SF-12 quality of life score at the end of the study. Cases of failure of treatment were considered as failure of pain relief (both effective and complete), and their scores (Izbicki, SF-12) were calculated at the last time they were assessed. All the scores differed enormously from the baseline (p<0.001, Mann-Whitney u-test). There was no difference concerning pain and quality of life at the time of follow-up between the Frey and the extended Partington groups.

**Figure 2:**
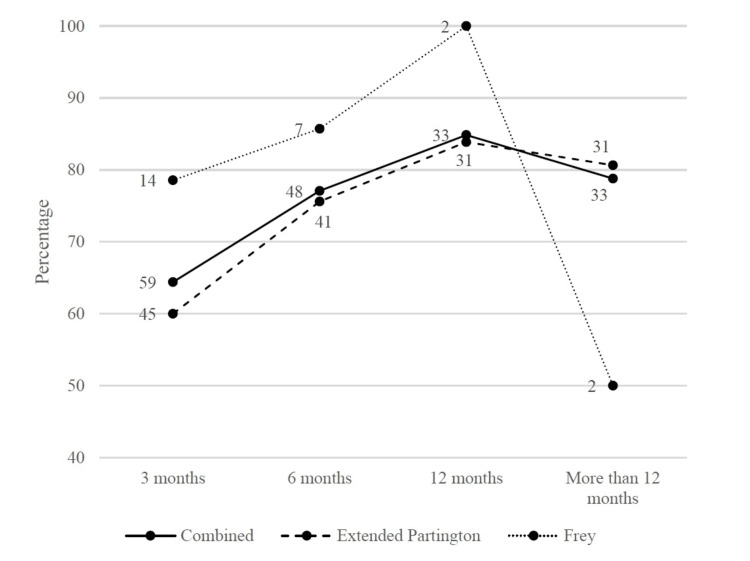
Trend in effective pain relief rate during the follow-up time The number of observations is shown next to each marker.

**Figure 3:**
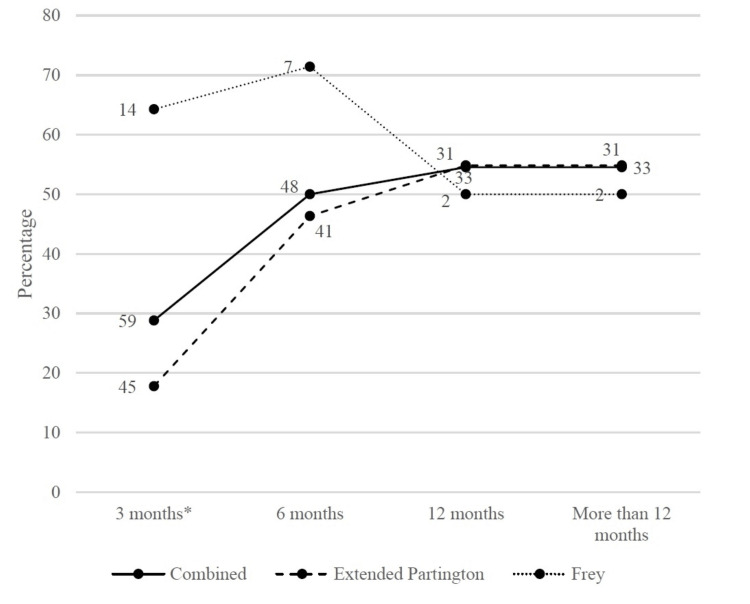
Trend in complete pain relief rate during the follow-up time The number of observations is shown next to each marker. * There is a statistically significant difference between the Frey group and the extended Partington group, using the Chi-square test.

**Figure 4:**
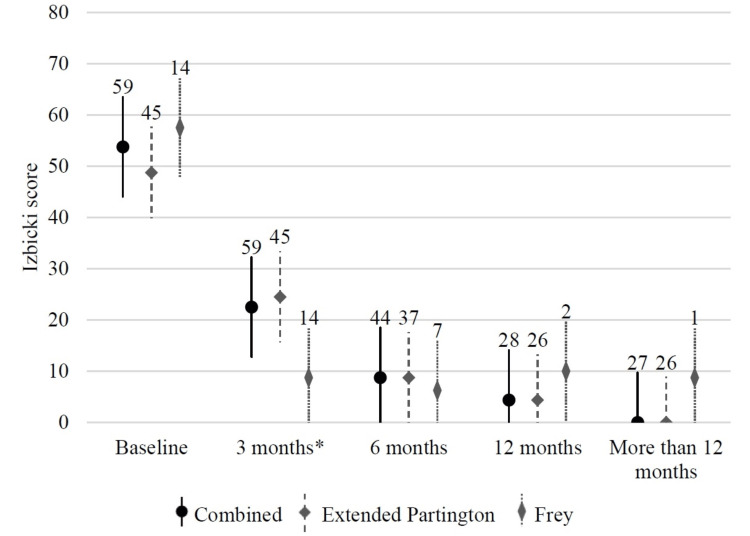
Trend in Izbicki score during the follow-up time Bars represent standard errors. The number of observations is shown above each bar. * There is a statistically significant difference between the Frey group and the extended Partington group, using Mann-Whitney u-test.

Figure 2 and Figure 3 depict the trend in effective pain relief and complete pain relief rates. Pain relief improved gradually up to 12 months, then stabilized. At three months, complete pain relief was gained much more in the Frey group than in the extended Partington group. Figure 4 depicts the trend in the Izbicki score. The score decreased rapidly in the first six months, then fell slowly. At three months, the Izbicki score was significantly lower in the Frey group than in the extended Partington group.

Pancreatic function and other manifestations

The exocrine and endocrine insufficiencies did not change much at the end of the study compared to the baseline. Nausea, anorexia, and weight loss were less observed than the baseline. Weight gain was seen more in the Frey group than in the extended Partington group (Table 3).

**Table 3:** Pancreatic function and other manifestations after follow-up * There are statistically significant differences between the time of follow-up and the baseline, using the Chi-square test. ** There is a statistically significant difference between the time of follow-up and the baseline, using the Fisher exact test.^ a ^There is a statistically significant difference between the Frey group and the extended Partington, using the Fisher exact test.

Variables	Combined	Extended Partington	Frey
Exocrine insufficiency	28 (48%)	24 (53%)	4 (29%)
Diabetes mellitus	19 (32%)	16 (36%)	3 (21%)
Insulin dependant	12 (20%)	10 (22%)	2 (14%)
Dose of insulin (IU)	25.5 (SD: 8.7)	26 (SD: 8.9)	23 (SD: 9.9)
Sufficiency persisted	37 (63%)	26 (58%)	11 (79%)
Insufficiency resolved	4 (7%)	4 (9%)	0 (0%)
Insufficiency persisted	10 (17%)	9 (20%)	1 (7%)
Insufficiency developed	8 (14%)	6 (13%)	2 (14%)
Weight (kg)	52.7 (SD: 9.5)	52.2 (SD: 8.9)	54 (SD: 11.4)
BMI (kg/m^2^)	19.7 (SD: 2.7)	19.7 (SD: 2.5)	19.5 (SD: 3.4)
Nausea*	8 (14%)	7 (16%)	1 (7%)
Anorexia*	10 (17%)	9 (20%)	1 (7%)
Weight loss**	3 (5%)	2 (4%)	1 (7%)
Weight stay	46 (78%)	38 (84%)	8 (57%)
Weight gain ^a^	10 (17%)	5 (11%)	5 (36%)
Diarrhea	9 (15%)	8 (18%)	1 (7%)

## Discussion

The limits of the original Partington procedure were mentioned in the literature. Prinz et al. [18] reported 14 cases of redrainage of the duct after an original Partington procedure. The operative findings confirmed that the ducts in the head had not been drained fully. After complete redrainage of the ducts, pain relief was gained in 71%. Adams et al. [19] intended to open the entire length of the duct completely; however, they failed to incise the duct in the head area in some cases due to severe stricture. They thought that part of the duct in the pancreatic head was the most probable source of recurrent pain. Isaji [6] concluded that 50% of patients developed recurrent pain after an original Partington procedure. This occurred because segments of the duct in the pancreatic head, which often contained stones, were not drained completely. O’Neil and Aranha [11] modified the original Partington procedure, in which an additional incision into the head of the pancreas is made, which is called the extended Partington procedure. Ho and Frey [10] suggested using the Frey procedure, which includes coring out of the pancreatic head, even in patients without an inflammatory mass in the head to ensure complete duct clearance. Isaji [6] suggested using both extended Partington and Frey procedures for patients whose inflammatory process happened in the pancreatic head. A consensus was reached regarding using both Frey and extended Partington procedures in chronic pancreatitis with a normal-sized pancreatic head and a dilated duct. However, the level of evidence is still weak [8].

Studies that directly compared Partington and Frey procedures are rare. In a study by Sakata et al. [5], they divided patients into modified Frey and minimum Frey (similar to extended Partington). The results of pain relief, pain score, and reoperation rate due to recurrent pain were not different between the two groups. Paye et al. [20] compared the results of the Frey and Partington procedure, but by analyzing this paper, we recognized that they compared the extended Partington and the original one. The results showed that pain relief was more favorable in the extended group. van der Gaag et al. [21] found that the reoperation rate due to recurrent pain or biliary obstruction was higher in the resection group than in the drainage group. They supposed that the Partington procedure should be preferred to the Frey procedure in patients without an enlarged pancreatic head. van der Gaag et al. [21] did not declare the extended drainage technique. Kempeneers et al. [22] are some of the scarce authors who described the extended drainage technique in detail. Instead of using the Partington procedure, they used the extended lateral pancreaticojejunostomy (i.e., the extended Partington procedure) and made some comparisons with the Frey procedure. The complication rate was significantly higher in the Frey group. There was no difference between the two groups concerning the pain and quality of life outcomes. Kempeneers et al. [22] suggested that the Frey procedure was unnecessary for patients without an inflammatory mass in the head.

In the early period of this current study, we attempted to perform the extended Partington procedure virtually in all patients. However, in some patients whose pancreatic heads were densely fibrotic and severely calcified, the complete removal of stones was impossible by purely incising the duct. Given the better ability of stone removal, the Frey procedure was used more in the late period of the study. The complication rate in the Frey group was higher but not significantly (21% vs. 4%). At three months, the complete pain relief rate and the Izbicki score were more favorable in the Frey group than in the extended Partington group, presumably because of more complete duct clearance. The quality of life was similar between the two groups at all time points. In contrast to van der Gaag et al. [21] and Kempeneers et al. [22], we thought the Frey procedure should be applied even to patients with a normal-sized head of the pancreas, especially those with densely fibrotic pancreatic heads.

The Frey procedure is concerned with worsening the pancreatic function compared to the extended Partington. Sakata et al. [5] did not find a difference in pancreatic function between the modified Frey and extended Partington. van der Gaag et al. [21] showed that endocrine insufficiency was seen more in the resection than in the drainage group. Still, their resection group included the majority of enlarged pancreatic heads. The current study did not find a difference in pancreatic function between the two groups. Furthermore, the Frey group had a higher rate of weight gain than the extended Partington. This shows that the Frey procedure has a similar capability of preserving pancreatic function compared to the extended Partington procedure in patients with a normal-sized head of the pancreas.

This study has some limitations. Firstly, the study was not designed as a randomized trial. Thus, the comparison between the two surgical techniques may be inaccurate. Secondly, our length of follow-up is somewhat insufficient since a minimum length of follow-up of five years is required to assess the long-term outcomes of surgical treatment for chronic pancreatitis. Thirdly, we lacked measurements of assessing exocrine pancreatic function such as fecal elastase-1 or fecal fat, thus the exocrine insufficiency was not defined well.

## Conclusions

The current study is one of the scarce studies that assessed the use of both Frey and extended Partington procedures in chronic pancreatitis patients with a normal-sized pancreatic head and a dilated duct. In this population, the use of Frey or extended Partington procedure is still controversial. The results showed that both procedures resulted in excellent pain relief and quality of life improvement without worsening the pancreatic function. The Frey procedure could yield a more favorable result regarding the pain in the early postoperative period than the extended Partington procedure. However, this study was not designed initially to compare the two procedures. Thus, a further randomized controlled trial should be conducted in the future.
